# Dietary exposure and health risk assessment of fumonisins through the consumption of maize and maize-based gluten-free products in individuals with celiac disease

**DOI:** 10.3389/fnut.2026.1672457

**Published:** 2026-01-27

**Authors:** Eylem Ezgi Tuyben, Sumeyra Sevim, Arife Macit, Mevlude Kizil

**Affiliations:** 1 Department of Nutrition and Dietetics, Hacettepe University, Ankara, Türkiye; 2 Department of Nutrition and Dietetics, Ankara Medipol University, Ankara, Türkiye; 3 Department of Nutrition and Dietetics, Munzur University, Tunceli, Türkiye

**Keywords:** exposure, fumonisin, gluten-free product, Monte Carlo simulation, risk assessment

## Abstract

**Introduction:**

Individuals with celiac disease (CD) must adhere to a gluten-free diet (GFD) and are therefore at high risk of fumonisin exposure due to their high consumption of maize and maize-based products. This study aimed to determine the concentrations of fumonisins (FB1, FB2, and FB3) in packaged gluten-free (GF) maize and maizebased products sold in Turkey and to assess dietary exposure and health risks in adults with CD using a probabilistic approach.

**Methods:**

Fumonisin concentrations of 51 GF products were analyzed by Enzyme-Linked Immonosorbent Assay (ELISA) and daily food consumption data were collected from 67 individuals with CD through a food frequency questionnaire. Exposure was estimated using Estimated Daily Intake (EDI) values and probabilistic risk assessment with Monte Carlo simulation.

**Results:**

The highest fumonisins concentrations were detected in the pasta/rice/bulgur group, while the flour/starch group was the food group contributed most to total fumonisins exposure due to higher consumption. Although the average EDI values were below the Tolerable Daily Intake (TDI; 1 μg/kg bw/day) set by European Food Safety Authority’s (EFSA) and Provisional maximum tolerable daily intakes (PMTDI; 2 μg/kg bw/day) established by Joint Expert Committee on Food Additives’s (JECFA) values, individuals at the 95^th^ percentile approached 132.88–167.89% of the PMTDI value in the flour/starch group and 79.00–82.38% in the pasta/rice/bulgur group.

**Discussion:**

These findings highlight the need for continuous monitoring and stricter regulatory oversight and dietary guidance to protect vulnerable populations.

## 1 Introduction

Fumonisins are a group of mycotoxins produced by Fusarium species, primarily *Fusarium verticillioides* and *Fusarium proliferatum* and classified into different series as A, B, C, and P in which the B-series (FB1, FB2 and FB3) are the most common mycotoxins found in maize and maize-based foods. Especially, FB1 is considered the main fumonisin due to its high occurrence (1).

Fumonisins have been associated with several adverse outcomes in epidemiological studies, and their toxicological effects are well documented in animal models. Moreover, fumonisin exposure has been linked with toxic effect on sensitive target organs such as liver and kidney (2). Previous studies have suggested that fumonisin B1, the most abundant fumonisin, may act as a potential etiological factor for certain human cancers, particularly esophageal and hepatocellular carcinoma, in populations with high maize consumption and inadequate cereal storage conditions (3, 4). Mechanistically, disruption of sphingolipid metabolism, increased oxidative stress, and dysregulated apoptosis and cell proliferation have been identified as potential pathways through which fumonisin exposure may promote carcinogenesis (4, 5). In this regard, individuals with CD often rely heavily on maize-based products as part of their gluten-free diet, which may increase their potential exposure. Hence, monitoring fumonisin concentrations in human foods remains essential to ensure food safety and to protect vulnerable populations. Moreover, due to its carcinogenic effect, The International Agency for Research on Cancer classified FB1 in Group 2B as a possible human carcinogen (6). In this regard, JECFA set a PMTDI value for FB1, FB2, and FB3, either alone or in combination of 2 μg/kg bw/day (7). However, EFSA established a TDI for FB1, FB2, FB3, and FB4 of 1 μg/kg bw (8).

Since fumonisins are found in many cereals, especially maize and maize based foods, the interest in these mycotoxins has been increased in recent years (9). Compared to other cereals and cereal-based foods, the highest occurrence and concentrations are in maize and its derivatives (10). In this regard, The European Union set maximum limits total fumonisins in maize and maize based foods. Accordingly, 1,000 μg/kg FB1 + FB2 was approved for “Maize and maize-based products intended for direct human consumption,” 800 μg/kg for “maize-based breakfast cereals and maize-based snacks,” and 200 μg/kg FB1 + FB2 was approved for “Processed maize-based foods and baby foods for infants and young children” (11, 12). The Turkish Food Codex also established the same limits (12). Furthermore, it was stated that fumonisins occurrence in uncooked maize and foods derived from maize with an average worldwide frequency of about 75 and 35%, respectively (10, 13). Therefore, dietary exposure to these mycotoxins has become a notable public health issue, particularly in some countries where maize consumption is a daily habit or due to dietary restrictions such as in individuals with CD.

CD is an immune-mediated, chronic disorder triggered by consumption of gluten containing grains in genetically predisposed individuals (14). Since CD is a lifelong condition, people with it are unable to consume any food that contains even trace amounts of wheat, oats, rye, barley, or their derivatives. In contrast, maize, rice, potatoes, and a few other cereals and pseudocereals are naturally GF foods widely used as carbohydrate sources by individuals with CD (15). People affected by CD therefore replace the carbohydrate source in their diet with maize or rice-based products. Hence, the daily intake of maize-based foods in individuals with CD could be much higher than in individuals without CD, and consequently their fumonisins intake could be significant (9).

In recent years, increasing attention has been given to assessing mycotoxin exposure by foods using deterministic and probabilistic approaches. The probabilistic approach provides a more realistic exposure estimate for a given population than deterministic approaches, considering the certain food consumption and food concentration data (1). Although global studies have addressed the risks of fumonisins exposure, comprehensive health risk assessment has not been studied specifically for adults with CD. In addition, with the increase in popularity and consumption of GF products in recent years, public health concern is highlighted to evaluate fumonisins exposure not only to individuals with CD but also the general population. To the best of our knowledge, this study is first to evaluate the risk assessment for fumonisins using consumption data of individuals with CD in Turkey. The aims of this study were to (i) analyze the presence of fumonisins (FB1, FB2, and FB3) in GF maize and maize-based packaged products sold in Turkey and to (ii) evaluate the dietary exposure and health risk assessment of fumonisins, which are the main mycotoxins posing health risks to individuals with CD.

## 2 Materials and methods

### 2.1 Sample collection

An exhaustive market survey was conducted to identify GF packaged maize and maize-based products available to consumers. A total of 51 products belonging to 14 different were purchased from grocery stores of various sizes (local markets, supermarkets, and hypermarkets) as well as from widely used online retailers in Ankara, Turkey in May 2024. The sampling strategy aimed to cover a broad range of product categories that are commonly consumed by individuals adhering to a gluten-free diet (GFD). The samples included 5 GF breads, 17 GF flours and starches for use in recipes (bread, cake), 6 GF pasta/rice/bulgur varieties, 5 GF breakfast cereals varieties, 9 GF crackers, grissini and chips, and 9 GF sweet biscuits and cookies. All products were verified as GF according to the labeling provisions of the Turkish Food Codex Regulation on Labeling Foods and Providing Information to Consumers (12). The collected samples were ground into flour with an average particle size of 50 μm, homogenized and stored at 20° C until analysis. All stored homogenized samples were analyzed within about 2 weeks. The sampling strategy aimed to include the most consumed GF maize-based products by individuals with CD, following a similar approach to Esposito et al. (9) and Brera et al. (14).

### 2.2 Determination of fumonisin concentration

The concentration of fumonisins (FB1, FB2 and FB3) were determined using the Bio-Shield Fumonisin, B2848/B2896, is an immunoassay method that determines the Fumonisins, in grains, cereals and other commodities including animal feed.

Chromatographic methods such as Liquid Chromatography–Tandem Mass Spectrometry (LC–MS/MS) are used for the detection of fumonisins; however, ELISA is widely preferred due to its cost-effectiveness and ease of application. Studies have shown that ELISA results were correlated with LC–MS/MS and HPLC and support their reliability in maize matrices (16–18). While acknowledging that chromatographic reference methods such as HPLC or LC–MS/MS provide higher analytical sensitivity and selectivity, it is also evident that ELISA provides reliable quantitative performance when properly validated and applied within its quantitation. According to numerous comprehensive reviews and validation studies (19–25), ELISA has been commonly recognized as quick, sensitive, specific, and quantitative analytical methods for routine determination of mycotoxins in food and feed. Moreover, these studies highlight that ELISA kits offer high throughput, cost-effectiveness, and consistency while producing results equivalent to chromatographic reference methods like HPLC or LC–MS/MS when used within the validated quantification range and with the proper calibration.

Specifically, Coronel et al. found no significant differences (*p* > 0.05) between the commercial ELISA and HPLC-FLD methods for fumonisin measurement in maize when concentrations were within the ELISA quantification range. They also found a highly positive association between the two methods. Only when analyte concentrations were higher than the top calibration limit or when samples were improperly diluted did differences up to twofold occur. Crucially, Coronel et al. and other researchers have already shown that ELISA tended to overestimate concentrations within the range and underestimate them above the range (16, 19, 20).

All analyses in our investigation were carried out within the manufacturer’s validated ELISA quantification range using duplicate determinations of homogenized composite samples from three retail units per product type. With this compound-pair design, we aim to minimize variability arising from both sampling and analytical processes. Furthermore, we sought to express fumonisin exposure not as a single deterministic value, but by taking into account possible overestimation and underestimation arising from kit-related factors, particularly through probability distributions (5th, mean, and 95th percentile intervals). In this way, we explicitly incorporated the uncertainty naturally arising from consumption and analytical data. For this reason, we would like to state that we do not consider it appropriate to discuss only the high consumer scenario. Therefore, while acknowledging that ELISA is an immunological testing technique with potential matrix-related interferences, our approach has ensured that the fumonisin concentrations obtained are quantitatively interpretable and scientifically robust within a defined analytical context by following validated procedures and uncertainty management principles.

The ELISA kit was applied according to the manufacturer’s instructions. A standard calibration curve (0–6 ppm) was used to determine fumonisins concentrations in food samples based on absorbance values. Absorbance was measured at 450 nm using a microplate reader (BioTek, Winooski, VT, United States). Calibration curves were generated using standard solutions included in the kit.

The analysis began with the extraction procedure for the products to be used, as specified in the manufacturer’s instructions. First, 20 g ground portion of samples were measured and added 100 mL of the Extraction Solvent (70% methanol) and mix in a blender for a minimum of 2 min. Allow the particulate matter to settle, filtered 5–10 mL of the extract through a Whatman #1 filter paper. After, collected the filtration and diluted 10 times with deionized water (example: 1 mL filtrate + 9 mL deionized water). The ratio of samples to 7% methanol is 1:50 (w/v). After diluting the obtained extract at a 1:10 ratio with 70% methanol in distilled water, the samples became ready for analysis.

For ELISA analysis, all reagents were brought to room temperature (19–24 °C) before use. First, 200 μL of Fumonisin Detection Solution was added to each dilution well. Then added 100 μL of each standard and prepared samples duplicate to the appropriate Dilution Well containing Fumonisin Detection Solution. Pipetted and mixed at least 5 times. Transferred 100 μL of content from each Dilution Microwell to the Antibody Coated Microtiter Wells in duplicate. The microwells were sealed with sealing film and incubated for 10 min at room temperature. The sealing film was then removed, and the liquid was drained from each well on the plate. Then 300 μL of Wash Buffer was added to each well with a multichannel micropipette and the plate was washed by manually shaking for a few seconds. This washing process was repeated four times. After this step, 100 μL of TMB Substrate was added to each well. The microwells were sealed again with sealing film, the plate was shaken by hand for a few seconds and incubated for 5 min at room temperature in the dark. After all these steps, removed the sealing film and added 100 μL of Stop Solution to each well. After adding the stop solution, absorbance was finally measured at 450 nm on a microplate reader (BioTek, Winooski, VT, United States). The fumonisins concentration was calculated from a calibration curve obtained using five fumonisins standard solutions at the following concentrations: 0.0, 0.15, 0.6, 2.4 and 6.0 ppm in 70% MeOH.

According to the manufacturer’s instructions, limit of detection (LOD) of 0.08 ppm and a limit of quantification (LOQ) of 0.12 ppm and the recovery rate of spiked samples was measured as 105% and the coefficient of variation (CV) was determined as 8.3%. For the validation method, three samples were tested by spiking 20 ± 0.05 g of the ground sample with fumonisin at concentrations within the range of the standard curve supplied with kit (12). Fumonisins mixture (FB1 and FB2) was acquired from Sigma–Aldrich (St. Louis, MO, United States). Each of the three GF biscuits was spiked with 0.0, 1.0, 3.0 and 5.0 ppm fumonisins. The extraction procedure and ELISA were carried out as detailed above. The average recovery rates for the fumonisin kit were found to be 84.2–112.6%, and the manufacturer’s recovery rate was within this range (105%).

### 2.3 Exposure analysis and health risk assessment

The exposure was determined using a deterministic method that divided food consumption by body weight and combined it with the fumonisins contamination value. Additionally, exposure calculations were conducted at the 95th percentile (P95) and 5^th^ percentile (P5) of consumption in order to assess the high-consumer and optimist scenarios, respectively.

The exposure dose of fumonisins was calculated using (26):


$$\mathrm{EDI}(\frac{\mathrm{\mu g}}{\mathrm{kg}}\mathrm{bw})=\frac{\mathrm{Cf}\times \mathrm{Cc}}{\mathrm{BW}}$$

EDI represents the estimated daily intake of fumonisins per kilogram body weight (μg/kg bw), *Cf* represents the average fumonisins concentration in the food group (μg/g), Cc represents the average daily per capita consumption of the respective food group (g) and BW represents the average body weight (kg bw).

The potential health risks that may arise from chronic exposure to total fumonisins have been assessed by comparing exposure percentages with the PMTDI values of 2.0 μg/kg bw per day established by JECFA.

Fumonisins exposure resulting from the consumption of maize and maize-based packaged products was estimated using a probabilistic approach based on Monte Carlo simulation techniques. The estimated daily intake (EDI, μg/kg/day) of fumonisins was derived by combining data on fumonisins concentrations in GF maize and maize-based packaged products with individuals’ food consumption amounts. The consumption data for GF maize and maize-based packaged products were obtained from food consumption frequency data of adults (n = 67) diagnosed with CD by a physician, and the fumonisins concentrations in the products were determined by ELISA. A log-normal distribution was applied to both input variables (fumonisins analysis values and individuals’ consumption quantities), and exposure values were simulated using random sampling. All simulations were performed using the R software (version 4.5.0; R Foundation for Statistical Computing, Vienna, Austria). To assess chronic exposure to fumonisins in food groups through diet, a probabilistic risk assessment was conducted using Monte Carlo simulations under three different consumption scenarios. Each scenario was simulated with 10,000 iterations to capture the variability of consumption patterns and contamination levels, and results were reported with 95% confidence intervals to ensure robustness and reproducibility of the risk estimates. The scenarios were developed to minimize uncertainty arising from consumption and analytical data based on the consumption characteristics of three demographic groups of individuals with CD:

Scenario 1 (General population): For all participants, exposure estimates at different levels were made by combining individual consumption data of all participants at their mean, 5th, and 95th percentile levels with fumonisin concentrations in the six food groups at their mean, 5^th^, and 95^th^ percentile levels.

Scenario 2 (Female participants): For female participants, exposure estimates at different levels were made by combining individual consumption data of female participants at their mean, 5th, and 95th percentile levels with fumonisin concentrations in the six food groups at their mean, 5^th^, and 95^th^ percentile levels.

Scenario 3 (Male participants): For male participants, exposure estimates at different levels were made by combining individual consumption data of male participants at their mean, 5th, and 95th percentile levels with fumonisin concentrations in the six food groups at their mean, 5^th^, and 95^th^ percentile levels.

Although mean, 5^th^ percentile exposure values were calculated to reflect the full distribution of the population, the risk characterization in this study was primarily based on the 95th percentile (high-consumer scenario) and high-consumer exposure estimates. A deterministic high-consumer scenario was calculated by multiplying, for each food group, the 95th percentile of daily consumption (g/day) with the 95^th^ percentile fumonisin concentration (μg/g, dry matter basis) and dividing by the mean body weight of the corresponding subgroup (female, male, or total population), and the resulting EDI₉₅ values (μg/kg bw/day) were compared with the provisional maximum tolerable daily intake (PMTDI) of 2 μg/kg bw/day established by JECFA (2001).

The Monte Carlo simulation was conducted for the three scenarios, using average consumption and fumonisin concentrations, with 10,000 iterations, to provide a probabilistic assessment of exposure. EDI values are expressed in μg/kg bw/day compared with the 1 μg/kg bw/day (EFSA, 2018) TDI threshold value to assess potential health risks.

### 2.4 Food consumption data

In our study, a Food Consumption Frequency Questionnaire (FCFQ) was developed to determine the consumption amounts and frequencies of GF maize and maize-based packaged products among individuals with CD. The questionnaire was developed based on studies in literature that evaluated the dietary habits of CP (22, 23). The food frequency questionnaire involved 6 groups: (i) Bread, (ii) Flour/starch, (iii) Pasta/rice/bulgur, (iv) Breakfast cereals, (v) Crackers/grissini/chips and (vi) Sweet biscuits/cookies. The questionnaire was applied face-to-face by the researchers to individuals with CD. The researchers also collected anthropometric data via measured body weight and height from the participants. The study was conducted under the Declaration of Helsinki, and ethical approval was obtained from Hacettepe University Health Sciences Research Ethics Committee (Approval number: SBA 24/1052). Informed consent was obtained from all participants.

The consumption amount of food groups was obtained by summing the consumption amounts of foods in each group, which represent Cc in the EDI formulas. Additionally, to take into consideration the heterogeneity of food consumption data, mean, 5th (low consumers), and 95th (high consumers) percentile consumption levels were computed for exposure analysis and health risk assessment. Moreover, the average body weight of the individuals participating according to anthropometric data in the study also represents bw in the EDI formula.

### 2.5 Fumonisin concentration data

The fumonisin concentration data from the analyses carried out for this investigation were utilized to calculate exposure. Each food analyzed was grouped according to the 6 food groups previously determined and the average fumonisin amounts of the food groups were calculated. These amounts represent *Cf* in the EDI formulas. Moreover, to take into consideration the heterogeneity of fumonisins concentration data means, 5th and 95th percentile concentration levels were also computed for exposure analysis and health risk assessment.

### 2.6 Statistical analysis

Statistical analyses were performed using SPSS software (version 22.0; IBM Corp., Armonk, NY, United States). Descriptive statistics were calculated for continuous variables; mean and standard deviation values were reported. Categorical variables were summarized using frequency and percentage (%) distributions.

To assess dietary exposure, a lower bound approach was applied. All concentrations below the LOD (0.08 ppm) were considered non-detected (ND) and assigned a value of zero for the calculations. Concentrations equal to or above the LOD (≥0.08 ppm), including those between the LOD and the LOQ (0.12 ppm), were included in the analysis without substitution or adjustment. For exposure and risk assessment, percentile values were provided corresponding to low (5th percentile), average (50th percentile), and high (95th percentile) consumption scenarios. The EDI of fumonisins mycotoxin from the diet for participants was modeled using a 10,000-iteration Monte Carlo simulation conducted with RStudio software (R Foundation for Statistical Computing, Vienna, Austria). The simulation outputs were presented as probability distributions; these values were compared with the TDI levels recommended by EFSA.

Since the purpose of this descriptive study was to estimate exposure rather than perform statistical difference analysis, no significance test was applied. However, a significance level of *p* < 0.05 was used as the threshold for all analyses.

## 3 Results

### 3.1 Demographic characteristics of individuals with celiac disease

The demographic characteristics of individuals participating in this study are presented in Table 1. Of the individuals with CD, 41 (61.2%) were female and 26 (38.8%) were male. The mean age of the individuals was 33.46 ± 12.71 (range: 19–64). In the present study, 13.43% of participants were classified as underweight, 55.22% as normal weight, 22.39% as overweight, and 8.96% as obese.

**Table 1:** Demographic and anthropometric characteristics and adherence to a gluten-free diet of individuals with celiac disease.

Characteristics of individuals	Female (*n* = 41)		Male (*n* = 26)		All participants (*n* = 67)	
Age (Mean±SD)	33.22 ± 12.32		33.84 ± 13.55		33.46 ± 12.71	
Waist-to-Hip Ratio (Mean ± SD)	0.81 ± 0.07		0.84 ± 0.07		0.82 ± 0.07	
	n	%	n	%	n	%
Body Mass Index (BMI)						
Underweight (<18.5)	4	9.76	5	19.23	9	13.43
Normal weight (18.5–24.9)	27	65.85	10	38.46	37	55.22
Overweight (25–29.9)	6	14.63	9	34.62	15	22.39
Obese (≥30)	4	9.76	2	7.69	6	8.96
Adherence to a GFD						
Always	32	78.05	12	46.15	44	65.67
Often	9	21.95	12	46.15	21	31.34
Sometimes	0	0.0	1	3.85	1	1.49
Rarely	0	0.0	1	3.85	1	1.49
Never	0	0.0	0	0.0	0	0.0

BMI, body mass index; GFD, gluten-free diet; SD, standard deviation.

Regarding adherence to the GFD, 65.67% of participants reported always adhering to the diet, while 31.34% reported adhering to it frequently. Although this high adherence rate is encouraging, the fact that GFD is of vital importance for individuals with CD should not be overlooked. It is important to remember that a significant proportion of individuals with CD may unknowingly or unintentionally be exposed to gluten, which can exacerbate the adverse health effects of the disease and prevent mucosal healing in the intestines.

### 3.2 Consumption amounts and fumonisins contents of gluten-free food groups

This study presents current and comprehensive data on the consumption habits of individuals with CD in Turkey regarding GF maize and maize-based products. As shown in Table 2, the highest mean daily consumption was observed in the flour/starch group (146.57 g/day), followed by bread (75.98 g/day) and pasta/rice/bulgur groups (69.02 g/day). In terms of high-consumer intake (95th percentile), flour/starch products reached 508.59 g/day in the total population (379.97 g/day in females and 550.78 g/day in males), indicating substantial variability and frequent use of these products. Similarly, bread consumption reached 206.86 g/day (156.86 g/day in females and 260.81 g/day in males), and pasta/rice/bulgur intake peaked at 216.05 g/day (196.20 g/day for females and 234.75 g/day for males). The consumption amounts for breakfast cereals, crackers/grissini/chips, and sweet biscuits/cookies were found to be lower compared to other food groups.

**Table 2:** Daily consumption amounts (g/day) of gluten-free food groups of individuals with celiac disease.

Food groups	Consumption amounts (g)								
	Female			Male			Total		
	Mean	5%	95%	Mean	5%	95%	Mean	5%	95%
Bread group	66.88	0.27	156.86	90.34	0.82	260.81	75.98	0.93	206.86
Flour/starch group	131.72	7.74	379.97	169.99	11.63	550.78	146.57	11.77	508.59
Pasta/rice/bulgur group	58.75	0.25	196.20	85.21	8.75	234.75	69.02	1.00	216.05
Breakfast cereals	15.43	0.00	102.62	29.96	0.00	223.09	21.07	0.00	103.62
Crackers/grissini/chips group	36.63	0.00	109.49	39.97	0.58	216.92	37.93	0.00	113.14
Sweet biscuits/cookies group	26.44	0.00	92.07	46.83	0.00	273.43	34.35	0.00	126.29

P5/P95, 5th/95th percentile.

In this study, fumonisins were analyzed in 51 GF maize and maize-based packaged products. The 51 products analyzed were grouped under six main food categories. Each product was assigned a code based on its brand and product type. The study revealed that products in the breakfast cereals and pasta/rice/bulgur groups had the highest average fumonisins concentrations (0.25 μg/g), while products in the bread group had the lowest fumonisins concentrations (0.10 μg/g; Table 3).

**Table 3:** Concentration of fumonisin (FB1, FB2, FB3) in gluten-free maize and maize-based foods (μg/g, dm).

Food groups	Fumonisins concentration
Bread group	**0.1010 ± 0.0857**
B1B1	0.216 ± 0.0857
B1B2	0.182 ± 0.0857
B1B3	ND
B2B1	ND
B3B1	0.107 ± 0.0857
Flour/starch group	**0.1901 ± 0.1141**
B1FS1	0.147 ± 0.1141
B1FS2	0.145 ± 0.1141
B1FS3	0.42 ± 0.1141
B4FS1	0.278 ± 0.1141
B4FS2	0.167 ± 0.1141
B4FS3	0.122 ± 0.1141
B5FS1	ND
B5FS2	0.0802 ± 0.1141
B6FS1	0.133 ± 0.1141
B7FS1	0.192 ± 0.1141
B7FS2	0.244 ± 0.1141
B8FS1	0.216 ± 0.1141
B2FS1	0.261 ± 0.1141
B2FS2	0.448 ± 0.1141
B2FS3	0.124 ± 0.1141
B9FS1	0.131 ± 0.1141
B3FS1	0.125 ± 0.1141
Pasta/rice/bulgur group	**0.2518 ± 0.1615**
B1PRN1	0.175 ± 0.1615
B10PRN1	0.149 ± 0.1615
B11PRN1	0.197 ± 0.1615
B12PRN1	0.229 ± 0.1615
B9PRN1	0.577 ± 0.1615
B9PRN2	0.184 ± 0.1615
Breakfast cereals group	**0.2476 ± 0.0714**
B1BC1	0.22 ± 0.0714
B1BC2	0.194 ± 0.0714
B8BC1	0.098 ± 0.0714
B9BC1	0.367 ± 0.0714
B13BC1	0.359 ± 0.0714
Crackers/grissini/chips group	**0.2017 ± 0.1148**
B1CGC1	0.218 ± 0.1148
B1CGC2	0.346 ± 0.1148
B6CGC1	0.172 ± 0.1148
B6CGC2	0.139 ± 0.1148
B13CGC1	0.148 ± 0.1148
B13CGC2	0.108 ± 0.1148
B13CGC3	0.216 ± 0.1148
B8CGC1	0.256 ± 0.1148
B9CGC1	0.212 ± 0.1148
Sweet biscuits/cookies group	**0.1422 ± 0.1751**
B1SBC1	0.106 ± 0.1751
B6SBC1	0.364 ± 0.1751
B14SBC1	ND
B14SBC2	ND
B13SBC1	ND
B9SBC1	ND
B1SBC2	ND
B1SBC3	0.166 ± 0.1751
B1SBC4	0.529 ± 0.1751

ND, not detected; dm, dry matter. Sample codes were created by combining the brand number (B) with the abbreviation of the corresponding food group (e.g., SBC = sweet biscuits/cookies; FS, flour/starch; PRN, pasta/rice/bulgur; BC, breakfast cereals; CGC, crackers/grissini/chips; B, bread). If more than one product from the same brand was available in a given food group, they were further distinguished by sequential numbering (e.g., B1FS1, B1FS2). All fumonisin concentrations are expressed as μg/g on a dry matter basis. Fumonisin concentrations (<LOD = 0.08 ppm) are indicated as ND and were treated as zero in calculations. Bold values represent the average of Fumonisins concentraton in each product group.

### 3.3 Dietary risk assessment for fumonisins

**Figure 1:**
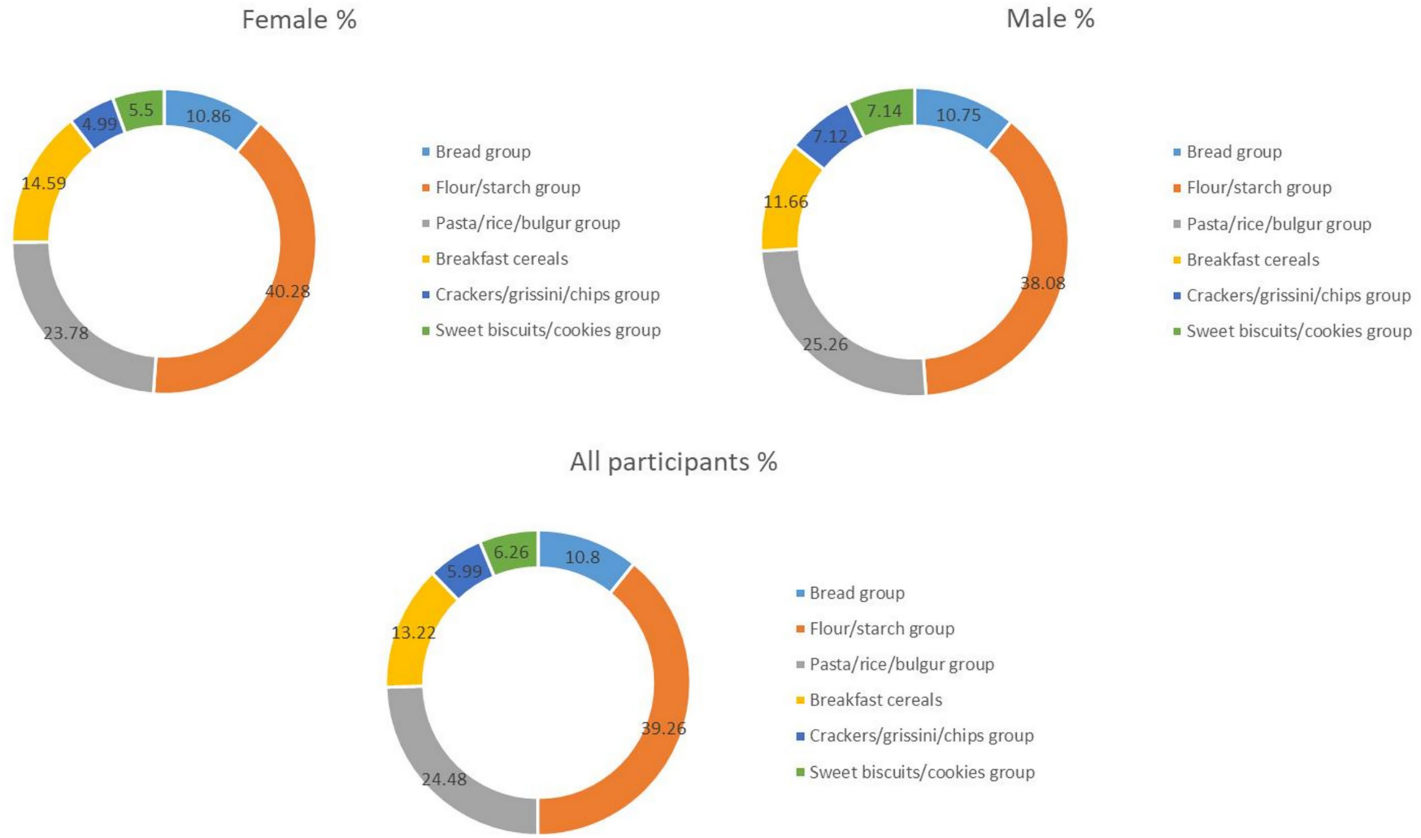
Percentage contribution of gluten-free food groups to chronic dietary intake of fumonisins among female, male, and all participants. Contribution (%) of different GF food categories to chronic dietary fumonisin intake. Relative contributions of each GF food group to total chronic dietary fumonisin exposure. GF, gluten-free.

The contribution of the consumption levels of the six food groups to chronic fumonisins intake for the general population, women, and men is detailed in Figure 1. According to the findings of our study, the average exposure values for all groups were generally below the TDI. However, it was determined that the flour/starch group was the group contributing most to exposure for all participants (39.26%), followed by the pasta/rice/bulgur group. The same food groups also contributed to fumonisins exposure in similar proportions according to gender (Figure 1). The contribution of the sweet biscuits/cookies and crackers/grissini/chips groups to exposure was found to be minimal (Figure 1).

Table 4 presents the EDI and percentage of the PMTDI for fumonisins across six gluten-free food groups. When compared with the PMTDI of 2 μg/kg bw/day, bread, breakfast cereals, crackers/grissini/chips, and sweet biscuits/cookies showed low exposure at mean intake levels, with values generally below 25% of the PMTDI in both genders.

**Table 4:** Chronic dietary exposure to fumonisins and associated risk assessment in individuals with celiac disease (μg/kg bw/day).

Food groups	Female		Male		Total	
	EDI (5–50-95%)	PMTDI%	EDI (5–50-95%)	PMTDI%	EDI (5–50-95%)	PMTDI%
Bread						
Mean	0.11 (0.02–0.23)	13.02	0.13 (0.02–0.27)	18.86	0.12 (0.02–0.25)	16.25
5%	0.00	2.00	0.00	2.89	0.00	2.49
95%	0.26 (0.04–0.54)	26.96	0.38 (0.06–0.78)	39.07	0.32 (0.05–0.67)	33.65
Flour/starch						
Mean	0.41 (0.14–0.92)	59.38	0.46 (0.16–1.04)	75.03	0.43 (0.15–0.97)	75.22
5%	0.02 (0.01–0.05)	20.04	0.03 (0.01–0.07)	25.32	0.04 (0.01–0.08)	25.39
95%	1.19 (0.40–2.66)	132.88	1.50 (0.51–3.36)	167.89	1.50 (0.51–3.37)	168.32
Pasta/rice/bulgur						
Mean	0.24 (0.15–0.47)	40.59	0.31 (0.19–0.60)	42.33	0.27 (0.17–0.53)	42.30
5%	0.00	25.07	0.03 (0.02–0.06)	26.14	0.00	26.12
95%	0.81 (0.50–1.58)	79.00	0.85 (0.52–1.65)	82.38	0.85 (0.52–1.65)	82.32
Breakfast cereals						
Mean	0.15 (0.07–0.19)	22.28	0.14 (0.07–0.18)	38.46	0.15 (0.07–0.18)	21.78
5%	0.00	10.83	0.00	18.70	0.00	10.59
95%	0.45 (0.22–0.56)	27.89	0.77 (0.37–1.17)	48.16	0.44 (0.21–0.55)	27.27
Crackers/grissini/chips						
Mean	0.05 (0.03–0.09)	17.01	0.09 (0.05–0.16)	32.22	0.07 (0.04–0.12)	16.25
5%	0.00	9.87	0.00	18.69	0.00	9.43
95%	0.34 (0.20–0.62)	30.78	0.65 (0.37–1.17)	58.32	0.33 (0.19–0.59)	29.41
Sweet biscuits/cookies						
Mean	0.06 (0.00–0.20)	9.79	0.09 (0.00–0.31)	25.34	0.07 (0.00–0.25)	12.71
5%	0.00	0.00	0.00	0.00	0.00	0.00
95%	0.20 (0.00–0.70)	35.03	0.51 (0.00–1.81)	90.67	0.25 (0.00–0.91)	45.47

EDI, Estimated Daily Intake; PMTDI, Provisional Maximum Tolerable Daily Intake (2 μg/kg bw/day, JECFA, 2001); bw, body weight. P5/ P95, 5^th^ and 95^th^ percentiles of intake. EDI values are presented as mean and 5^th^–95^th^ percentiles based on individual consumption and measured fumonisin concentrations. PMTDI (%) values were calculated using the 95^th^ percentile EDI for each food group to better reflect high-consumer exposure compared with the JECFA PMTDI of 2 μg/kg bw/day.

However, exposure markedly increased at the 95th percentile (high-consumer scenario). In the bread group, 95^th^ percentile values reached 26.96% in females, 39.07% in males, and 33.65% in the total population. Similar trends were observed for breakfast cereals (27.89–48.16-27.27% PMTDI) and crackers/grissini/chips (30.78–58.32-29.41% PMTDI). Although these groups did not exceed the PMTDI, they contributed non-negligibly to overall exposure among high-consuming males. In contrast, the pasta/rice/bulgur group exhibited substantially higher exposure, with mean values of 40.59–42.33% PMTDI and 95th percentile values reaching 79.00% (females), 82.38% (males), and 82.32% (total), indicating that frequent consumption can approach health-based guidance values. The flour/starch group represented the most critical exposure source. Mean PMTDI values were 59.38% (females), 75.03% (males) and 75.22% (total population), while 95^th^ percentile exposure exceeded the safety threshold, reaching 132.88, 167.89, and 168.32%, respectively. This confirms that high consumers of gluten-free flour and starch-based products may surpass the PMTDI. Gender-related differences were evident, with males consistently exhibiting higher PMTDI percentages due to greater consumption quantities. While mean exposures across all groups remained below the PMTDI, reliance only on average values would underestimate risk, particularly for individuals with high intake of flour/starch and pasta/rice/bulgur products.

Overall, these findings indicate that average dietary exposure to fumonisins does not pose a critical health risk; however, high consumers—especially males and individuals regularly consuming gluten-free flour and starch products—may reach or exceed health-based guidance values. This highlights the necessity for continuous monitoring, stricter control of mycotoxin contamination, and dietary risk assessment in sensitive populations such as individuals with CD.

**Figure 2:**
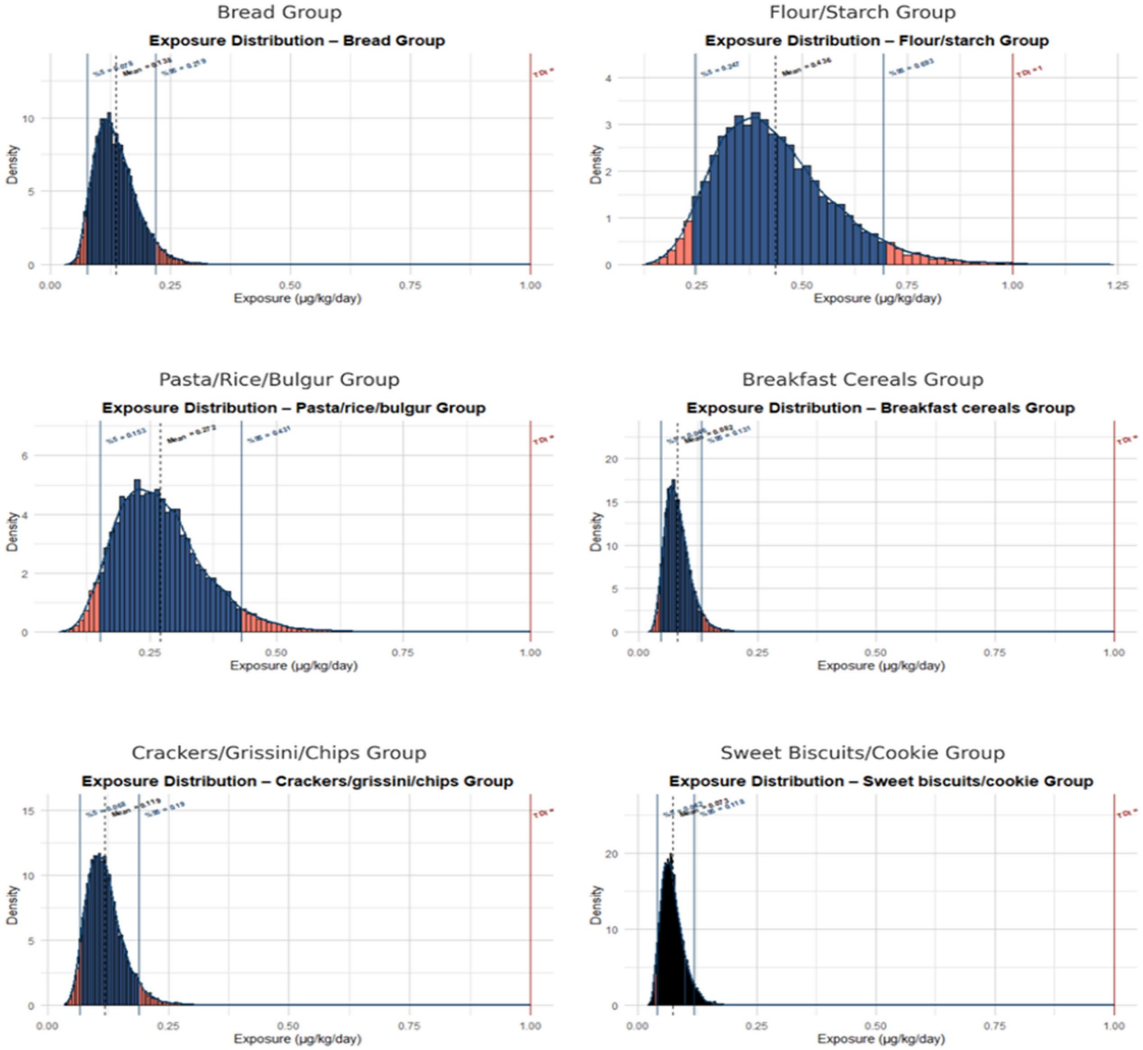
Probability distributions of chronic fumonisin exposure among the total population for the six food groups. Monte Carlo simulation (10,000 iterations) of chronic dietary fumonisin exposure based on consumption and concentration variability. Vertical red line indicates the EFSA tolerable daily intake (TDI, 1 μg/kg bw/day).

Monte Carlo simulations across the three scenarios highlighted clear differences in fumonisin exposure by gender and product group. Scenario 1 (Figure 2), representing the total study population, provided the baseline exposure profile. Scenario 2 (Supplementary Figure 1) reflected female consumption patterns, while Scenario 3 (Supplementary Figure 2) represented male participants, who generally showed higher exposures at the 95th percentile due to greater consumption.

## 4 Discussion

As shown in Table 1, the proportion of women was higher among the participants in this study (41 women and 26 men) that is consistent with global evidence indicating that CD is approximately 1.5 times more prevalent in women than in men, as confirmed by biopsy (27). Regarding BMI distribution, 13.43% of participants were underweight, 22.39% overweight, and 8.96% obese. These rates closely align with the meta-analysis by Barone et al. (2023) on over 15,000 CD patients, which reported prevalence of 11.04% underweight, 18.42% overweight, and 11.78% obese (28). While our study showed a slightly higher proportion of overweight individuals and a slightly lower proportion of obese individuals, the overall trend supports previous reports indicating a shift in the clinical profile of CD, with increasing prevalence of overweight and obesity in addition to the classical malnutrition phenotype (29). Taken together with other studies, these findings demonstrate that CD is no longer characterized solely by thinness. This change has been attributed to earlier diagnosis and greater awareness of atypical or silent forms.

As shown in Table 2, the mean daily consumption values of all participants across the six food groups were 75.98 g for bread, 146.57 g for flour/starch, 69.02 g for pasta/rice/bulgur, 21.07 g for breakfast cereals, 37.93 g for crackers/grissini/chips, and 34.35 g for sweet biscuits/cookies. The flour/starch group (146.57 g/day) was mostly consumed GF products. Considerable interindividual variability was observed, with the 95th percentile intake reaching 508.59 g/day for flour/starch, 206.86 g/day for bread, and 216.05 g/day for pasta/rice/bulgur. Compared with European data, some differences were observed. Valitutti et al. reported average daily intakes of 36.5 g/day for bread, 53.3 g/day for pasta, 27.2 g/day for biscuits, and 14.9 g/day for crackers (30). Similarly, in a multicenter study conducted in Europe (Germany, Norway, Italy, and Spain), Gilbert et al. reported that the average daily bread consumption was highest in Northern European countries such as Germany and Norway, at 128.7 g/day and 126.8 g/day, respectively, followed by Italy at 68.5 g/day and Spain at 54.3 g/day. The same study noted that pasta consumption was highest in Italy at 64.7 g/day. Additionally, it was reported that products such as biscuits, crackers, and breakfast cereals consumptions were varied by country (31). Overall, our findings highlight both similarities and differences with European studies. The relatively high intake of flour/starch products among individuals with CD may be explained by differences in dietary habits, limited availability of gluten-free products, their accessibility, and individual preferences.

The concentrations of fumonisins in different GF maize and maize-based food groups are presented in Table 3. The highest concentration of fumonisins was observed in the pasta/rice/bulgur group (0.2518 μg/g), followed by breakfast cereals (0.2476 μg/g) and the flour/starch group (0.19 μg/g). In contrast, the bread group had the lowest mean concentration (0.10 μg/g). Among the six food groups analyzed, the sweet biscuits/cookies group had the widest range of fumonisins levels, varying from undetectable to a maximum of 0.51 μg/g. The findings highlight that, despite the results generally remaining below the international regulatory limits, certain food groups could significantly contribute to total fumonisins exposure to humans when consumed in high quantities on a regular basis.

In the high-consumer scenario, for each food group, the 95th percentile fumonisin concentration was multiplied by the 95^th^ percentile intake and divided by mean body weight. Under these conditions, flour/starch-based products reached 1.50 μg/kg bw/day EDI value in females (132.88% PMTDI), 1.50 μg/kg bw/day EDI value in males (167.89% PMTDI), and 1.50 μg/kg bw/day EDI value in the total population (168.32% PMTDI), thereby exceeding the 2 μg/kg bw/day PMTDI (JECFA, 2001). Pasta/rice/bulgur products reached 0.81–0.85 μg/kg bw/day EDI value (79.00–82.38% PMTDI), while sweet biscuits/cookies in males reached 0.51 μg/kg bw/day EDI value (90.67% PMTDI). These findings confirm that although mean exposures were below PMTDI, high consumers—particularly males—may approach or exceed health-based guidance values when consuming flour/starch-based products frequently.

Studies on fumonisins in GF products are quite limited and most of the paper focuses on traditional products such as corn, corn flour, cereals, pasta and bread. Several countries have conducted studies that have shown significant frequencies of fumonisin occurrence in corn and corn-based products, along with contamination levels exceeding the upper limits permitted by mycotoxin food control regulations (32–34). A recent study conducted in Turkey found fumonisin (FB1 + FB2) in (55.6%) of 54 corn-based product (corn flakes, popcorn, cornsnacks) samples at concentrations ranging from 18 to 5,055 ng/g. High levels (95%) of fumonisins were found in corn snack samples (21/22) and 9 of 22 corn snack samples, fumonisin concentrations exceeded the EU limit value (800 ng/g) (31, 35). On the other hand, in a study conducted in Brazil, a total of 212 products (popcorn and corn-based products) were analyzed, and free fumonisins were detected in all samples (100%) and it is reported that the total amount of fumonisins ranged from 7.2 to 2158.2 μg/kg and none of samples exceeded the maximum tolerated level of 2,500 μg/kg according to Brazilian Health Surveillance Agency (33).

Additionally, Brera et al. demonstrated in their studies that fumonisins exposure in individuals with CD, particularly in children, could range from 348 to 582 ng/kg/day, but noted that these values remained below the PMTDI (14). Similarly, the findings of the study are supported by a recent study conducted by Song et al. in Dalian, China, which revealed that exposure levels in children and adolescents exceeded the 95th and 99th percentile values of 2 μg/kg body weight/day PMTDI (36). These results highlight an important risk factor observed in our study. Although exposure levels to fumonisins are generally within acceptable limits, individuals at the upper end of the consumption distribution, particularly individuals with CD may face significant health risks with long-term consumption. In the Monte Carlo simulation results of the study, exposure values were observed to approach critical threshold values at the 95^th^ percentile (Figure 2). Similarly, a study conducted by Gilbert-Sandoval and colleagues using nixtamalized maize samples in Mexico reported that FB1 + FB2 exposure values exceeded the PMTDI value in 47% of male participants and 30% of female participants (37). Moreover, Esposito et al. highlighted instances where total dietary fumonisin intake exceeded EFSA’s temporary maximum limits by up to 150%, underlining the importance of further evaluation of exposure in GF consumers (9).

As shown in Table 4, the EDI of total fumonisins (FB1 + FB2 + FB3) in individuals with CD varied significantly across food groups, ranging from an average of 0.12 μg/kg bw/day (bread) to 0.43 μg/kg bw/day (flour/starch). Among these, flour- and starch-based products contributed the highest mean exposure, corresponding to 59.38% PMTDI in females, 75.03% in males, and 75.22% in the total population, followed by pasta/rice/bulgur (40.59–42.33% PMTDI) and bread (13.02–18.86% PMTDI). At the 95th percentile (high-consumer scenario), flour/starch-based products reached 1.19–1.50 μg/kg bw/day EDI value, corresponding to 132.88% (female), 167.89% (male), and 168.32% (total population) of the PMTDI, thereby exceeding the tolerable intake. In the pasta/rice/bulgur group, 95th percentile exposure values reached 79.00% (female), 82.38% (male), and 82.32% (total) of the PMTDI, while sweet biscuits/cookies in males reached 90.67% PMTDI (95^th^ percentile). These findings indicate that although average exposures remain within safe limits, high consumers—particularly of flour/starch products—may reach or exceed health-based guidance values.

In the present study, mean fumonisin exposure was influenced by both contamination levels and consumption habits. Similar findings were reported by Dall’Asta et al., where Italian CD patients had a mean intake of 0.395 μg/kg bw/day (approximately 20% PMTDI) from GF products. However, our 95^th^ percentile EDI values for flour/starch products (up to 1.50 μg/kg bw/day) were nearly four times higher and exceeded the PMTDI, demonstrating that mean exposure alone underestimates actual risk among high consumers (38). These findings indicate that high consumption of flour/starch products may represent a health risk with respect to fumonisin exposure. Similar concerns were reported in probabilistic risk assessments conducted in Brazil, Mexico, and other regions. Lopes et al. found that the probability of PMTDI exceedance in maize-based foods reached up to 95.6% in some regions during the second harvest season (39). Gilbert-Sandoval et al. reported exceedances in 47% of male and 30% of female consumers in Mexico (37), while Andrade et al. noted regional differences linked to processing methods and consumption habits (1). Though, these studies underscore the strong influence of climate, agricultural practices, and processing conditions on fumonisin contamination. Compared with these reports, our study identified lower exposure levels, likely reflecting the specific characteristics of packaged maize-based products consumed in Turkey. Nonetheless, these comparisons highlight the importance of considering geographical variability and production processes in mycotoxin risk assessment and support the need for region-specific control policies.

Significant variations in fumonisin exposure patterns were found using gender-specific probabilistic models. High consumers reached 1.19 μg/kg bw/day EDI value (132.88% PMTDI), while flour and starch-based items contributed the most to mean exposure among females (0.41 μg/kg bw/day EDI value, 59.38% PMTDI). However, the exposure from the same food group was much higher in males (0.46 μg/kg bw/day EDI value on average) and reached 1.50 μg/kg bw/day EDI value at the 95th percentile, which is equivalent to approximately 167.89% of the PMTDI. Pasta/rice/bulgur-based products were the second most significant contributors in both genderss; however, the absolute intake was higher in men. The need to take gender-specific consumption patterns into account when evaluating dietary exposure, especially for GF staple goods, is underscored by our findings.

Compared to other studies in the literature, this study not only aligns with previously reported concerns regarding the adverse health effects of fumonisins exposure but also highlights food group-specific risks. The detection of higher fumonisins concentrations in breakfast cereals and pasta/rice/bulgur products, combined with their high consumption rates, underscores the importance of population-specific risk assessments. Additionally, the data obtained highlights the need for stricter regulations, monitoring protocols, and interventions in the GF food industry.

The public health relevance of our findings is also highlighted. The safety of people on a GFD depends on regular monitoring the fumonisin concentrations in GF items and providing customers with appropriate labeling. Our results provide a basis for recommendations to reduce exposure to GF consumers by identifying food groups linked to increased exposure based on daily consumption habits. Moreover, for sensitive groups like individuals with CD, risk mitigation strategies including such as measures by food producers and regulatory authorities as well as consumer education and information campaigns are crucial. Briefly, these findings provide a framework for public health and recommendations for decision-makers in the fields of health and policy.

## 5 Conclusion

This study evaluated fumonisins (FB1, FB2, FB3) concentrations in GF maize and maize-based packaged products sold in Turkey and assessed potential health risk for individuals with CD who regularly consume these products in their diets. This study represents the first probabilistic risk assessment specifically conducted on GF maize and maize based packaged products frequently consumed by individuals with CD in Turkey. Overall, the study results indicate that the flour/starch and pasta/rice/bulgur groups contribute more to fumonisins exposure compared to other groups. Under high-consumption conditions, exposure from these groups approached or exceeded health-based guidance values, highlighting the importance of evaluating not only average consumers but also high-risk subgroups. Therefore, the need for stricter controls and regulations on the production processes of these two food groups is highlighted. In addition, the findings suggest that dietary diversification and clearer product labeling may help mitigate long-term exposure risks in sensitive populations. However, these findings should be interpreted with caution due to several limitations. First, the assessment of fumonisins exposure is based on self-reported consumption data, which may be subject to recall bias. Second, the fumonisin analysis was carried out solely by ELISA and not confirmed to chromatographic methods. Third, the sampling of GF maize-based products was limited to a specific time frame and market availability, which may not fully represent all products available in Turkey and the sample size of GF maize-based products was limited to a specific period and market availability. Additionally, the Monte Carlo simulation was based solely on data from adult individuals and did not include other vulnerable groups such as children and the elderly. Future research should incorporate larger and more diverse product datasets, including additional mycotoxins, use confirmatory analytical methods, and assess exposure in different demographic groups to provide a more comprehensive risk evaluation.

## Data Availability

The original contributions presented in the study are included in the article/Supplementary material, further inquiries can be directed to the corresponding author.
