# Supplementary material for: Dietary exposure and health risk assessment of fumonisins through the consumption of maize and maize-based gluten-free products in individuals with celiac disease
Source: Front Nutr. 2026 Jan 27;13:1672457. doi: 10.3389/fnut.2026.1672457 (PMC12886419; doi:10.3389/fnut.2026.1672457)
Supplement: Supplementary file 1 [file Supplementary_file_1.docx]

Supplementary Material

# Supplementary Figures and Tables

## Supplementary Figures

**Supplementary**
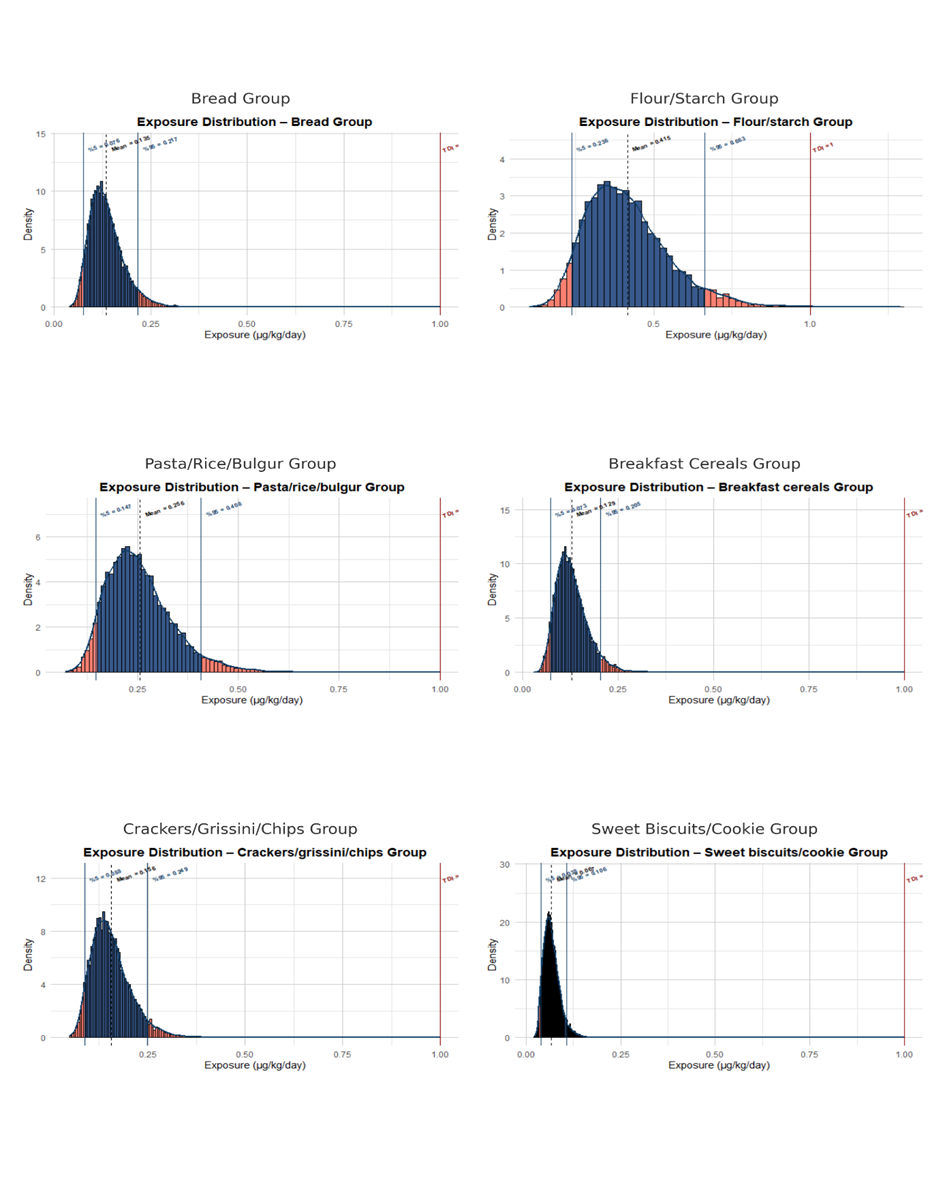
**Figure 1.** Probability distributions of chronic fumonisin exposure among the female population for the six food groups. Monte Carlo simulation (10,000 iterations) of chronic dietary

fumonisin exposure based on consumption and concentration variability.

Vertical red line indicates the EFSA tolerable daily intake (TDI, 1 µg/kg bw/day)


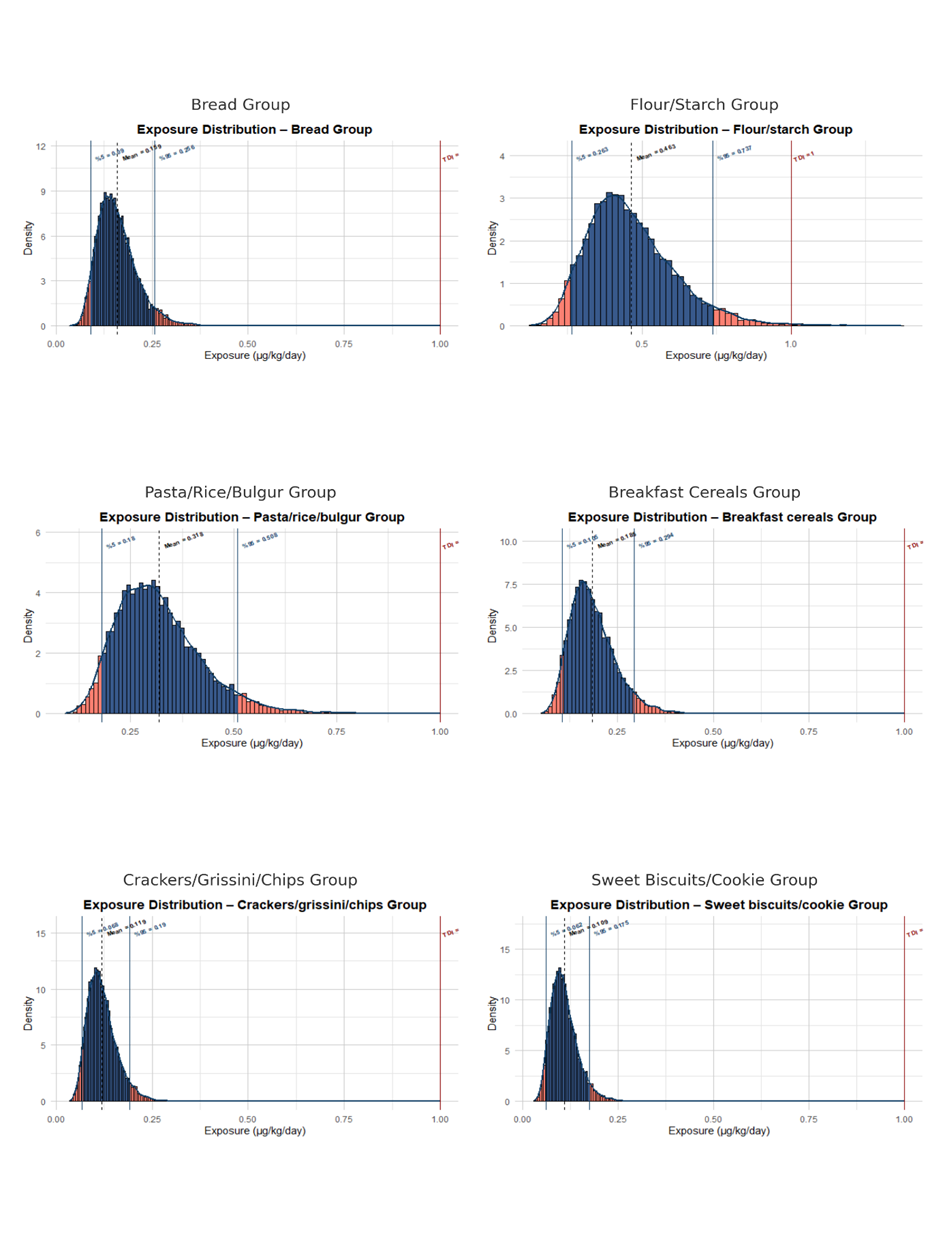
**Supplementary Figure 2.** Probability distributions of chronic fumonisin exposure among the male population for the six food groups. Monte Carlo simulation (10,000 iterations) of chronic dietary fumonisin exposure based on consumption and concentration variability.

Vertical red line indicates the EFSA tolerable daily intake (TDI, 1 µg/kg bw/day
